# The impact of bDMARDs on postoperative complications in patients with rheumatoid arthritis: A systematic review and meta-analysis

**DOI:** 10.1097/MD.0000000000036132

**Published:** 2023-11-24

**Authors:** Takahito Suto, Koichi Okamura, Hideo Sakane, Chisa Okura, Tetsuya Kaneko, Hirotaka Chikuda

**Affiliations:** a Department of Orthopaedic Surgery, Gunma University Graduate School of Medicine, Maebashi, Gunma, Japan; b Department of Orthopaedic Surgery, Fujioka General Hospital, Fujioka, Gunma, Japan; c Department of Orthopaedic Surgery, Gunma Saiseikai Maebashi Hospital, Maebaashi, Gunma, Japan; d Department of Orthopaedic Surgery, Japan Redcross Society Fukaya Redcross Hospital, Fukaya, Saitama, Japan.

**Keywords:** bDMARDs, postoperative complications, rheumatoid arthritis, SSI, VTE

## Abstract

**Background:**

The influence of biological disease-modifying antirheumatic drugs (bDMARDs) on postoperative surgical site infection (SSI) and venous thromboembolism (VTE) in patients with rheumatoid arthritis (RA) has not yet been clarified.

**Methods:**

A systematic literature search was performed using PubMed, Web of Science^TM^, Scopus, and The Cochrane Library databases to identify eligible studies published up to August 2023. All studies comparing postoperative SSI or VTE rates in RA patients with or without bDMARD treatment were included. The protocol for this study was registered in PROSPERO (CRD42021246264) and is available on the University of York website.

**Results:**

Overall, 20 studies with 71,885 RA patients and 6 studies with 7918 RA patients were included for postoperative SSI and VTE comparisons, respectively. Patients treated with bDMARDs had significantly higher rates of postoperative SSI than those without treatment (odds ratio 1.50, 95% confidence interval 1.23–1.83, *P* < .0001). However, these significant differences disappeared in the analysis restricted to 9 studies involving non-tumor necrosis factor α inhibitors. The use of bDMARDs seemed to increase the rate of postoperative VTE (odds ratio 2.20, 95% confidence interval 1.30–3.72, *P* = .003). A subgroup analysis showed that postoperative osseous complications were significantly less frequent in RA patients with bDMARD treatment than in those without treatment.

**Conclusion:**

RA patients treated with bDMARDs had an increased risk of not only postoperative SSI but also VTE. While bDMARD usage merits appropriate attention, there might be positive aspects as well. Further data will be needed to confirm the postoperative risks of bDMARD usage in RA patients.

**Key points:**

RA patients with bDMARD treatment have an increased risk of postoperative SSI and VTE.

## 1. Introduction

The treatment for patients with rheumatoid arthritis (RA) has improved dramatically over the past 20 years thanks to the development of biological disease-modifying antirheumatic drugs (bDMARDs), such as tumor necrosis factor (TNF) α inhibitors (e.g., infliximab, etanercept, adalimumab, golimumab, and certolizumab pegol), interleukin-6 inhibitors (e.g., tocilizumab), and cytotoxic T-lymphocyte-associated protein 4-immunoglobulin immunoconjugates (e.g., abatacept). These agents have markedly ameliorated the symptoms and improved the functional prognosis by delaying joint destruction in patients with RA. However, many patients still require orthopedic surgeries because of inevitable joint destruction despite the powerful therapeutic effects of these agents.

TNF released from macrophages is crucial for the formation and maintenance of granulomas as well as protection against invasion by intracellular organisms. Inhibition of TNF reportedly carries a risk of inducing a variety of infections in animal models and also in RA patients, as TNF α plays an important role in host immunity. Therefore, TNF α inhibitors are expected to have some influence on the rate of surgical site infection (SSI) in RA patients. However, while some reports have revealed the influence of bDMARDs on SSI in patients with RA, no consensus has yet been reached.^[1–5]^ The primary purpose of the present study was therefore to clarify whether or not bDMARDs carry a risk of increased SSI in RA patients undergoing orthopedic surgeries, especially now that more data has been accumulated.

Another major postoperative complication is venous thromboembolism (VTE), which includes both deep vein thrombosis and pulmonary embolism. Understanding postoperative VTE is essential to allow surgeons to manage their operations successfully, as VTE is a potentially life-threatening complication, especially in orthopedic surgeries, such as total knee arthroplasty (TKA) and total hip arthroplasty (THA). Since inflammatory environments are associated with endothelial dysfunction, RA patients may have an increased risk of developing VTE. Indeed, some cohort studies have shown an increased risk of VTE in patients with RA.^[6]^ However, contradictory results have been shown in a few studies comparing the influence of RA and osteoarthritis (OA) on VTE development after orthopedic surgeries.^[7]^ However, few reports have evaluated RA patients treated with bDMARDs, and the consensus regarding the risk of postoperative VTE in such patients is unclear.

Therefore, the present systematic literature review and meta-analysis were conducted to compare the risk of postoperative SSI and VTE in RA patients with and without bDMARD treatment.

## 2. Materials and methods

The protocol has been registered in the international Prospective Register of Systematic Reviews (PROSPERO) database (CRD42021246264). It is available in full on the University of York website. This study does not require ethical approval because the data will be obtained from already published studies.

### 2.1. Search strategy

**Figure 1.**
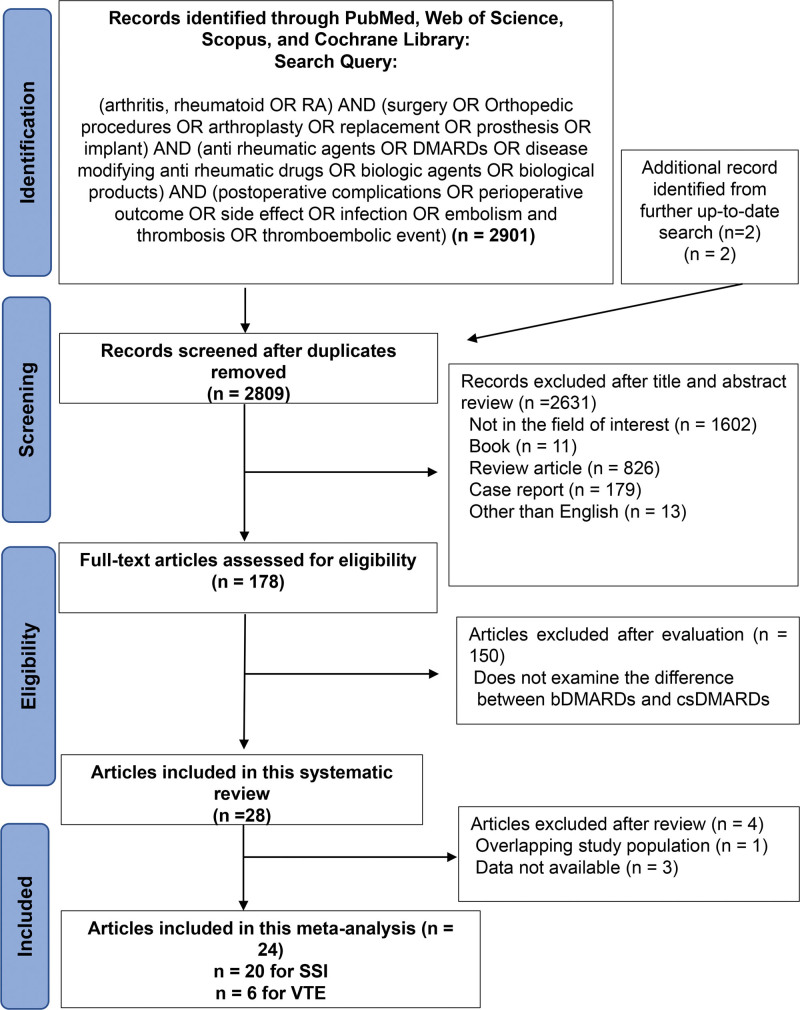
Flow diagram of the study selection procedure for the systematic review and meta-analysis. DMARDs = disease-modifying antirheumatic drugs, bDMARDs = biological DMARDs, csDMARDs = conventional synthetic DMARDs, SSI = surgical site infection, VTE = venous thromboembolism.

We performed the present study in accordance with the guidelines of the preferred reporting items for systematic reviews and meta-analyses (PRISMA) statement.^[8]^ A comprehensive literature search of the electronic databases PubMed, Web of Science, Scopus, and the Cochrane Library was performed on August 29, 2023, to investigate literature that focused on postoperative complications, including SSI and VTE, in RA patients using bDMARDs and conventional synthetic DMARDs (csDMARDs). All full-text papers were reviewed based on the study title and abstract. After this screening, full-text papers were assessed and excluded when found to be inappropriate. Two reviewers (TS and KO) independently conducted all searches, and differences were resolved by discussion. The search strategy is shown in Figure 1.

The search terms used in the present study were (“arthritis, rheumatoid” OR “RA”) AND (“surgery” OR “Orthopedic procedures” OR “arthroplasty” OR “replacement” OR “prosthesis” OR “implant”) AND (“anti rheumatic agents” OR “DMARDs” OR “disease modifying anti rheumatic drugs” OR “biologic agents” OR “biological products”) AND (“postoperative complications” OR “perioperative outcome” OR “side effect” OR “infection” OR “embolism and thrombosis” OR “thromboembolic event”).

### 2.2. Inclusion and exclusion criteria

Based on the PRISMA guidelines, we referenced the population, intervention, comparator, outcome, and study design to determine the inclusion criteria.^[8]^ We identified studies that referred to patients with RA who had undergone orthopedic surgeries and compared the impact of bDMARDs on the postoperative outcomes to that of all other medications. We did not ask about the continuation or discontinuation of bDMARDs. Studies containing Janus kinase inhibitors, which are the latest drug class of DMARDs showing comparable efficacy to TNF α inhibitors, were excluded because the mode of action may be different from bDMARDs. Studies included in this review were limited to cohort or observational studies with appropriate controls using non-bDMARDs.

We excluded reviews, editorials, meeting abstracts, replies from authors, letters to the editor, case reports, studies not published in English, and studies in which data were not obtainable. If multiple articles were published by the same group based on similar patient cohorts, only the most recent study or the largest one was incorporated into the analysis.

### 2.3. Data extraction

The information was extracted by 2 authors independently from the eligible articles. The information contained the following characteristics: author names, publication year, recruitment country, period of patient recruitment, follow-up period, number of patients, study design, medications for RA (type of bDMARDs and csDMARDs), type of surgery, and adverse events (SSI, VTE, and osseous complications). Data from each article are summarized in Tables 1 and 2.

**Table 1:** Baseline characteristics of the included studies for postoperative SSI.

Study, study design, country	Recruitment year	Patients, age, female (%)		Kind of medication		Type of surgery	Follow up (post surgery)	Definition of outcome
		BIO(+)	BIO(−)	BIO	Except BIO			
Bibbo et al,^[1]^ Prospective cohort, USA	Not specified	16 50 (44–66) Over 90%	15 60 (41–73) Over 90%	IFX, ETN	LEF, MTX, PSL	Foot and ankle surgery	BIO(+)/BIO(−) 10.6/ 9.7 mo	Infectious and healing complications, Unclear
Giles et al,^[2]^ Questionnaire survey, USA	1999–2004	35 Not specified	56 Not specified	TNFα inhibitor	HCQ, LEF, MTX, SSZ	Arthroplasty, fusion and resection, small joint procedure, revision	30 d	Wound infections, Clear
den Broeder et al,^[3]^ Retrospective cohort, Netherlands	1997–2004	104, 54 ± 16, 75% (stopped) 92, 57 ± 13, 82% (continued)	1023 61 ± 13 77%	IFX, ETN, ADA	PSL, MTX	Wrist and hand, ankle and foot, knee and hip surgery	1 yr	Superficial or deep SSI, Clear (1992 CDC*)
Bongartz et al,^[9]^ Retrospective cohort, USA	1996–2004	50 Not specified	412 Not specified	IFX, ETN, ADA, Anakinra	AZT, LEF, PSL, MTX	THA, TKA	>1 yr (mean 4.3 yr)	Wound infections, Clear
Hirao et al,^[10]^ Case control study, Japan	Not specified	22 Not specified	22 Not specified	TCZ	BUC, DPA, MTX, PSL, SSZ	Shoulder arthroplasty, TEA, THA, TKA, TAA, foot and hand surgery	2 wk	Superficial or deep infection, Unclear
Hirano et al,^[11]^ Retrospective cohort, Japan	2004–2007	39 58.9 ± 9.0 82%	74 62.6 ± 9.1 88%	IFX, ETN	MTX, PSL	TKA, THA, TEA, ankle arthrodesis, TAA, TSA	4 wk	Wound infections, dehiscence, Clear
Kawakami et al,^[12]^ Retrospective case control, Japan	2004–2009	64 57.0 (51.8–64.0) 80%	64 57.0 (47.0–64.0) 80%	IFX, ETN	MTX, PSL, SSZ	TKA, THA, ORIF	Not specified	Superficial and deep SSI, Clear (1999 CDC†)
Galloway et al,^[13]^ Prospective cohort, UK	2001–2008	2689 Not specified	659 Not specified	IFX, ETN, ADA	Not specified	TSA, TEA, THA, TKA	3 yr	Septic arthritis, Clear
Momohara et al,^[14]^ Retrospective cohort, Japan	2005–2009	48 Not specified	372 Not specified	IFX, ETN, ADA, TCZ	BUC, DPA, MTX, MZR, PSL, SSZ, TAC	TKA, THA	Not specified	Superficial or deep SSI, Clear (1999 CDC†)
Suzuki et al,^[15]^ Questionnaire survey	2004–2008	3468 Not specified	56339 Not specified	IFX, ETN, ADA, TCZ, ABT	MTX, PSL, TAC	TKA, THA, spine surgery	Not specified	Superficial or deep SSI, Clear (1999 CDC†)
Johnson et al,^[16]^ Retrospective cohort, USA	2007–2011	92 58.7 ± 12.4 84%	143 64.4 ± 10.5 90%	IFX, ETN, ADA, GLM	DMARDs, PSL	TKA	6 mo	Superficial or deep SSI, Clear (1992 CDC*)
Scherrer et al,^[17]^ Retrospective cohort, Switzerland	2000–2008	122 Not specified	1207 Not specified	IFX, ETN, ADA	DMARDs, PSL	Arthroplasties and osteosyntheses including arthrodesis	< 2 yr	Superficial or deep SSI, Clear (1992 CDC*)
Kubota et al,^[4]^ Retrospective cohort, Japan	2006–2011	267 59.2 ± 11.2 Not specified	300 65.8 ± 10.8 Not specified	IFX, ETN, ADA, TCZ, ABT	MTX, PSL	TKA, THA, TEA, TAA, TSA, total finger arthroplasty, toe arthroplasty	>1 yr	Superficial or deep SSI, Clear (1999 CDC†)
Kadota et al,^[5]^ Retrospective cohort, Japan	2004–2012	196 59 (28–80) Not specified	840 64 (15–88) Not specified	IFX, ETN, ADA, GLM, TCZ, ABT	AUF, BUC, MTX, PSL, SSZ, TAC	Hand and wrist, foot and ankle, spine, TKA, THA, TEA, TSA, arthroscopic synovectomy	Not specified	SSI, Clear (1999 CDC†)
Tada et al,^[18]^ Retrospective cohort, Japan	2006–2013	101 Not specified	231 Not specified	IFX, ETN, ADA, TCZ, ABT	MTX, PSL, SSZ, TAC	Orthopaedic surgery	>1 yr	Superficial or deep SSI, Clear (1999 CDC†)
Cordtz et al,^[19]^ Retrospective cohort, Denmark	2000–2014	345 61.3 ± 12.0 71%	1601 65.2 ± 10.7 74%	IFX, ETN, ADA, CZP, TCZ	MTX, PSL	TKA, THA	1 yr	Prosthetic joint infection, Unclear
Onodera et al,^[20]^ Retrospective cohort, Japan	2007–2016	17 Not specified	30 Not specified	Not specified	MTX, PSL	Forefoot surgery	12–114 mo	SSI, Unclear
Borgas et al,^[21]^ Retrospective cohort, Sweden	2006–2015	157 Not specified	237 Not specified	IFX, ETN, ADA, GLM, CZP	AZT, HCQ, LEF, MMF, MTX, PSL, SSZ	TKA, THA	1 yr	Superficial or deep SSI, Clear (1992 CDC*)
Ito et al,^[22]^ Retrospective case control, Japan	2011–2014	97 64.1 ± 10.8 Not specified	97 64.9 ± 9.7 Not specified	ABT	MTX, PSL, DMARDs	Spine, foot and ankle, shoulder and elbow, wrist and fingers	>1 yr	SSI, Clear (1992 CDC*)
Yamashita et al,^[23]^ Retrospective cohort, Japan	1997–2011	22 Not specified	120 Not specified	TNFα inhibitor	MTX, PSL	TKA	10.6 yr	Prosthetic joint infection, Unclear

ABT = abatacept, ADA = adalimumab, AUF = auranofin, AZT = azathioprine, BIO = biological agent, BUC = bucillamine, CDC = Centers for Disease Control and Prevention, CZP = certolizumab pegol, DPA = D-penicillamine, ETN = etanercept, GLM = golimumab, HCQ = hydroxychloroquine, IFX = infliximab, LEF = leflunomide, MMF = mycophenolate mofetil, MTX = methotrexate, MZR = mizoribine, ORIF = open reduction and internal fixation, PSL = prednisolone, SSI = surgical site infection, SSZ = sulfasalazine, TAA = total ankle arthroplasty, TAC = tacrolimus, TEA = total elbow arthroplasty, THA = total hip arthroplasty, TKA = total knee arthroplasty, TNF α = tumor necrosis factor α, TSA = total shoulder arthroplasty.

* SSI using Centre for Disease Control (CDC) criteria^[24]^.

† SSI using CDC criteria^[25]^.

**Table 2:** Baseline characteristics of the included studies for postoperative VTE.

Study, study design, country	Recruitment year	Patients, age, female (%)		Kind of medication		Type of surgery	Follow up (post surgery)	Definition of outcome
		BIO(+)	BIO(−)	BIO	Except BIO			
Bibbo et al,^[1]^ Prospective cohort, USA	Not specified	16 50 (44–66) Over 90%	15 60 (41–73) Over 90%	IFX, ETN	LEF, MTX, PSL	Foot and ankle surgery	BIO(+)/BIO(−) 10.6/ 9.7 mo	DVT, unclear
Kawakami et al,^[12]^ Retrospective case control, Japan	2004–2009	45 58.0 (53.0–63.0) 76%	45 59.0 (52.0–66.0) 76%	IFX, ETN	MTX, PSL, SSZ	TKA, THA, ORIF	Not specified	VTE using ultrasonography, Clear
Davies et al,^[26]^ Prospective cohort, UK	2001-	4572 Not specified	1012 Not specified	IFX, ETN, ADA	Not specified	TSA, TEA, THA, TKA	90 d	VTE, Clear
Johnson et al,^[16]^ Retrospective cohort, USA	2007–2011	92 58.7 ± 12.4 84%	143 64.4 ± 10.5 90%	IFX, ETN, ADA, GLM	DMARDs, PSL	TKA	6 mo	PE by computed tomography angiogram, DVT by Doppler scan or angiogram, clear
Cordtz et al,^[19]^ Retrospective cohort, Denmark	2000–2014	411 61.5 ± 11.6 72%	1391 65.3 ± 10.4 74%	IFX, ETN, ADA, CZP, TCZ	MTX, PSL	TKA, THA	1 yr	VTE, clear
Ito et al,^[22]^ Retrospective case control, Japan	2011–2014	88 Not specified	88 Not specified	ABT	MTX, PSL, DMARDs	Spine, foot and ankle, shoulder and elbow, wrist and fingers	>1 yr	DVT or PE, Unclear

ABT = abatacept, ADA = adalimumab, BIO = biological agent, DVT = deep venous thrombosis, ETN = etanercept, GLM = golimumab, IFX = infliximab, LEF = leflunomide, MTX = methotrexate, ORIF = open reduction and internal fixation, PE = pulmonary embolism, PSL = prednisolone, SSZ = sulfasalazine, TCZ = tocilizumab, TEA = total elbow arthroplasty, THA = total hip arthroplasty, TKA = total knee arthroplasty, TSA = total shoulder arthroplasty, VTE = Venous thromboembolism.

### 2.4. Statistical analyses

The main outcomes of the present meta-analysis were the assessment of the proportion of patients with RA who experienced postoperative SSI and VTE under the usage of bDMARDs or csDMARDs. The odds ratios (ORs) and 95% confidence intervals (CIs) were calculated for all comparisons. We used forest plots to assess the individual studies and obtained summary ORs of the value of postoperative SSI and VTE in RA patients with or without bDMARD treatment. To assess osseous complications suspected to be related to bDMARDs, subgroup analyses for complication rates were conducted. Heterogeneity among the outcomes of included studies was evaluated using the *I*^2^ statistic and the Cochran Q test. Significant heterogeneity was considered at *P* < .05 using the Cochrane Q test and a ratio >50% for *I*^2^ statistic, which led to the use of random effect models according to the DerSimonian and Laird method.^[27]^ We used fixed effect models for low-heterogeneity results. Publication bias was evaluated by a visual inspection of funnel plots for all of the assessed comparisons.

All statistical analyses were performed with the Review Manager 5.3 (The Nordic Cochrane Centre, Copenhagen, Denmark) statistics software program.

### 2.5. Risk of bias

The risk of bias for all prospective or retrospective cohort studies was evaluated with the Risk of Bias In Non-randomized Studies of Interventions (ROBINS-I).^[28]^ Two authors independently assessed the risk of bias in each study (Supplementary Tables 1–3, http://links.lww.com/MD/K775, http://links.lww.com/MD/K776, http://links.lww.com/MD/K777). All discrepancies regarding the risk of bias were resolved by consensus among coauthors.

## 3. Results

### 3.1. Study selection and characteristics

There were 2901 publications identified through PubMed, Scopus, and Web of Science and the Cochrane Library, and an additional 2 records were identified from a further up-to-date search. Overall, 2903 publications were identified for the initial assessment. After the elimination of duplicates, articles unrelated to the field of interest, books, reviews, case reports, and non-English text, a full text review was performed in 178 articles. Based on the selection criteria, we identified 24 articles for systematic review and a meta-analysis to assess differences in SSI and VTE rates, respectively.^[1–5,9–17,19–23,26,29–34]^ The selection process and list are shown in Figure 1. Extracted data and characteristics of the 20 studies that evaluated postoperative SSI are outlined in Table 1; in the same way, 6 studies describing VTE are listed in Table 2.

### 3.2. The comparison of postoperative SSI rates between RA patients with and without bDMARD treatment

**Figure 2.**
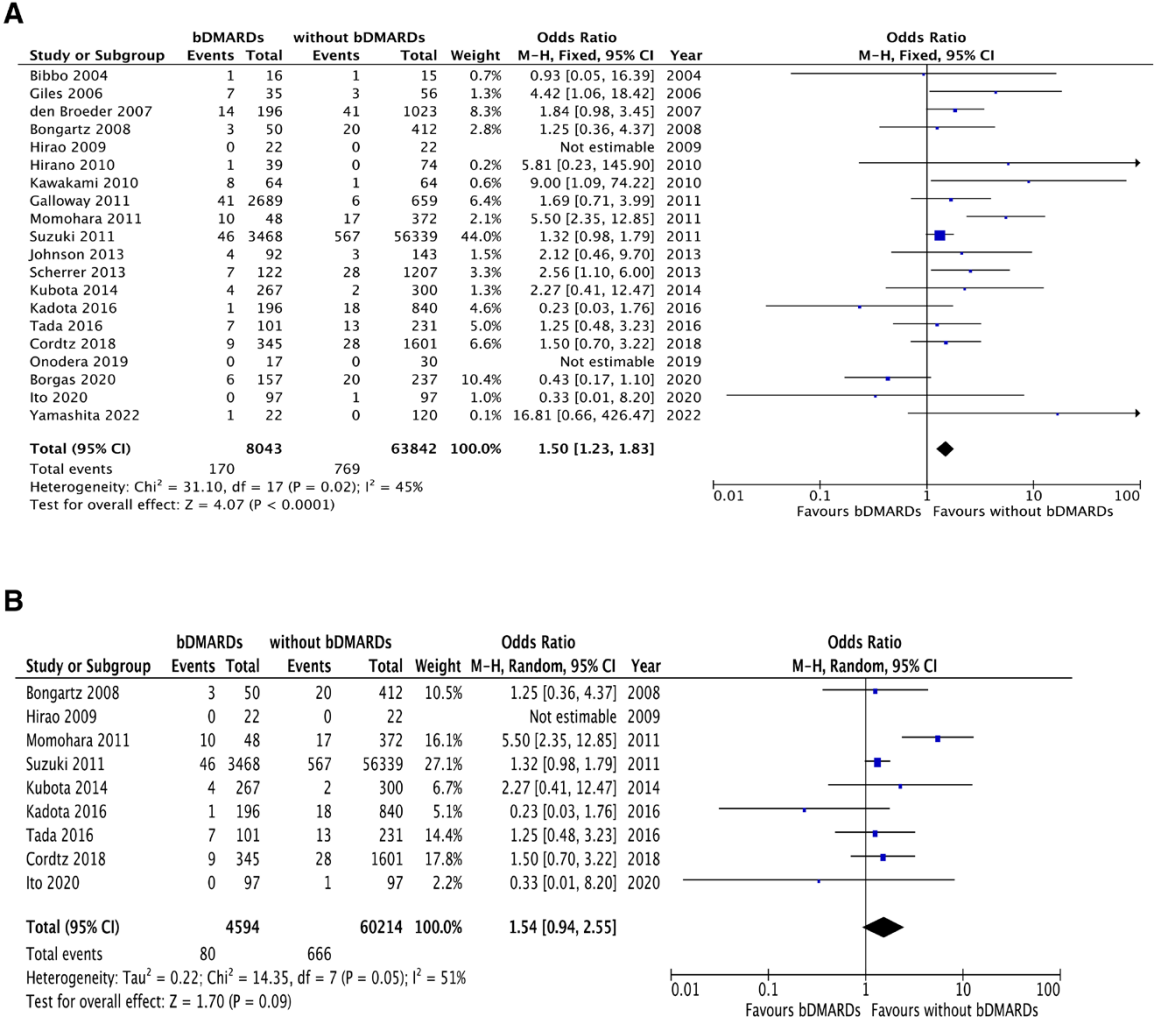
(A) Forest plots of studies investigating the comparison of postoperative SSI between RA patients with and without bDMARD treatment. (B) Analysis restricted to studies including non-TNF inhibitors. bDMARDs = biological disease-modifying antirheumatic drugs, RA = rheumatoid arthritis, SSI = surgical site infection, TNF = tumor necrosis factor.

We assessed the association between postoperative SSI and bDMARD use in 20 studies including 71,885 patients with RA. The forest plots (Fig. 2A) revealed that patients treated with bDMARDs had a significantly higher rate of postoperative SSI than those without bDMARD treatment (OR 1.50, 95% CI 1.23–1.83, *P* < .0001). The Cochran Q test (chi-square 31.10, *P* = .02) and I^2^ test (45%) showed low-heterogeneity significantly. The funnel plot identified 1 study over the pseudo 95% CI (Supplemental Fig.1, http://links.lww.com/MD/K778). Risk of bias for the primary outcome was generally considered moderate (Supplemental Table 1, http://links.lww.com/MD/K775).

### 3.3. Postoperative SSI after bDMARD treatment among studies involving non-TNF α inhibitors

Among 20 studies assessing postoperative SSI, 11 were conducted in RA patients treated with bDMARDs that did not include non-TNF α inhibitors, such as TCZ and ABT. Non-TNF α inhibitors are relatively new among bDMARDs products, so we incorporated the remaining 9 studies including non-TNF α inhibitors into the analysis of postoperative SSI.

In this analysis, 64,808 RA patients were assessed, and no significant differences were observed between the bDMARDs and non-bDMARDs groups (OR 1.54, 95% CI 0.94–2.55, *P* = .09) according to forest plots (Fig. 2B). The Cochran Q test (chi-square 14.35, *P* = .05) and I^2^ test (51%) showed significant heterogeneity.

### 3.4. The comparison of postoperative VTE rates between RA patients with and without bDMARD treatment

**Figure 3.**
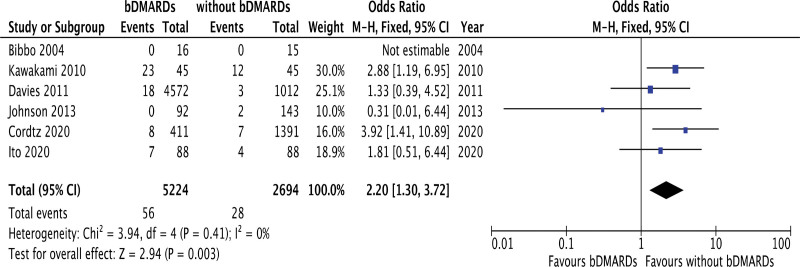
Forest plots of studies investigating the comparison of postoperative VTE between RA patients with and without bDMARD treatment. bDMARDs = biological disease-modifying antirheumatic drugs, RA = rheumatoid arthritis, VTE = venous thromboembolism.

The impact of bDMARDs on postoperative VTE was investigated in 6 studies including 7918 RA patients. Our meta-analysis showed that the odds ratio of bDMARDs was 2.20 with a 95% CI of 1.30–3.72 and *P* = .003. The forest plots are shown in Figure 3. The Cochran Q test (chi-square 3.94, *P* = .41) and I^2^ test (0%) were not significantly heterogeneous. Funnel plots did not demonstrate any studies over the 95% CI (Supplemental Fig.2, http://links.lww.com/MD/K779). According to this analysis, the rate of postoperative VTE was significantly higher in patients using bDMARDs than in those not using bDMARDs. More than half of studies had moderate risk of bias (Supplemental Table 2, http://links.lww.com/MD/K776).

### 3.5. A subgroup analysis of postoperative osseous complications between RA patients with and without bDMARD treatment

**Figure 4.**
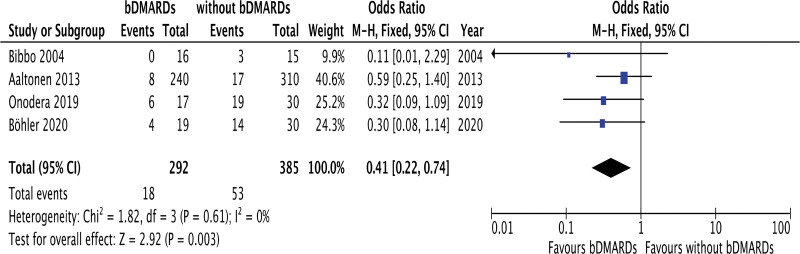
Subgroup analysis of postoperative osseous complications between RA patients with and without bDMARD treatment. bDMARDs = biological disease-modifying antirheumatic drugs, RA = rheumatoid arthritis.

To confirm the influence of postoperative osseous complications, a subgroup analysis was performed. Four studies including 677 RA patients investigated postoperative osseous complications, including loosening of implants, nonunion, and delayed union. In this analysis, most of the operations were TKA, THA, and other joint replacement procedures. According to the forest plots (Fig. 4), the presence of postoperative osseous complications was significantly lower in RA patients with bDMARD treatment than in those without it (OR 0.41, 95% CI 0.22–0.74, *P* = .003). The Cochran Q test (chi-square 1.82, *P* = .61) and I^2^ test (0%) indicated no significant heterogeneity. Funnel plots did not demonstrate any studies over the 95% CI (Supplemental Fig.3, http://links.lww.com/MD/K780). Serious risks of bias were detected in most of studies (Supplemental Table 3, http://links.lww.com/MD/K777).

## 4. Discussion

In this meta-analysis, we compared the risks of complications that mainly follow orthopedic surgeries, including SSI, VTE, and osseous complications, in RA patients treated with bDMARDs versus those without bDMARD treatment. Recently, an increasing number of RA patients are being treated with bDMARDs because of their efficacy, including RA patients who require orthopedic surgeries. Therefore, it is important for surgeons to reach a certain consensus regarding these postoperative complications when performing surgeries on patients with RA in the era of biological treatment. Although several reviews have focused on the postoperative SSI risk of bDMARDs, with some showing a trend toward an increasing risk in cases with bDMARD treatment, data are far from sufficient at present to answer this question.^[35–37]^ Our work showed that RA patients treated with bDMARDs are at a higher risk of postoperative SSI than those without such treatment (OR = 1.50, [1.23–1.83], *P* < .0001). Compared to the meta-analysis published recently, the present study is a much larger meta-analysis of 8021 RA patients treated with bDMARDs. The 2022 ACR guideline for patients with rheumatic diseases undergoing elective THA or TKA recommended withholding all current bDMARDs prior to surgery and scheduling the surgery for the end of the dosing cycle,^[38]^ leading to the widely recognized perception that bDMARDs can be a risk factor for infection. This recommendation seems to be consistent with the results of the current study.

One interesting aspect of the present study is that different results were obtained when we analyzed only those studies containing non-TNF α inhibitors, such as TCZ or ABT. Compared to TNF α inhibitors, non-TNF α inhibitors are relatively new, with fewer types available. Therefore, fewer studies have reported on infections caused by non-TNF α inhibitors. Our study is characterized by the fact that a greater number of patients using non-TNF α inhibitors were covered than in previous reports. We planned to exclude studies without non-TNF α inhibitors in order to understand more about the effect of non-TNF α inhibitors on postoperative SSI. As a result, it was demonstrated that there were no significant differences between the bDMARDs and non-bDMARDs groups, suggesting that non-TNF α inhibitors have less of an impact on postoperative SSI than TNF α inhibitors. Further analyses of data concerning non-TNF α inhibitors should be conducted in order to draw a more precise conclusion.

We focused on postoperative VTE in the present study because it is a potentially life-threatening complication after orthopedic surgery, including THA and TKA. Several previous large cohort studies have demonstrated that the risk of VTE was over 2-fold higher in RA patients than in the general population.^[6]^ Systematic chronic inflammatory conditions, such as RA, may change the thrombotic responses and lead to endothelial dysfunction. Consequently, there seems to be a certain view that patients with RA are at a high risk of VTE. However, the risk of postoperative VTE in patients with RA remains controversial. The incidence of VTE after TKA was compared in RA and OA patients in several previous studies, showing contradictory results.^[7,39]^ A meta-analysis of VTE after TKA in RA versus OA patients concluded that the VTE risk after primary TKA was lower in RA patients than in OA patients.^[40]^ Conversely, when considering RA patients, it was suggested that there was no significant difference in the VTE risk between those using bDMARDs and those using csDMARDs.^[41]^

The present study investigated the effect of bDMARDs on VTE after orthopedic surgeries, not limited to TKA. In our meta-analysis, the incidence of VTE after any orthopedic surgeries was significantly higher in RA patients with bDMARD treatment than in those without it. (OR = 2.20, [1.30–3.72], *P* = .003). Whether this result was caused by the bDMARDs themselves or an issue on the part of the patients, who had chronic inflammation to the extent that they had to use bDMARDs, is unclear. While more research is needed, our results are considered important for helping orthopedic surgeons performing surgeries on patients treated with bDMARDs in their daily clinical practice.

Orthopedic surgeries are always accompanied by postoperative problems, such as infections and VTE as well as implant loosening and the occasional need for revision. However, a clear consensus regarding these characteristic complications of orthopedic surgeries associated with bDMARDs has yet to be obtained. Therefore, subgroup analyses were performed in the current study. Aseptic loosening, nonunion, delayed union, revision, and floating of the lesser toes after resection arthroplasty were included among postoperative osseous complications.^[1,20,29,30]^ Our data demonstrated that postoperative osseous complications were less likely to occur in RA patients using bDMARDs than in those not using bDMARDs. Furthermore, RA patients receiving bDMARDs had markedly lower rates of aseptic loosening than RA patients under traditional DMARD treatment, as systemic inflammation in RA might influence local inflammatory osteolysis and can lead to aseptic loosening, which is the most common cause for surgical revision after TKA and THA.^[30]^ Taken together, these present and previous findings suggest that reducing inflammation and disease activity with bDMARD treatment is beneficial for RA patients requiring orthopedic surgeries.

Several limitations associated with the present study warrant mention. First, most of the studies included in this meta-analysis were retrospective cohort studies. The patient and surgeon preferences strongly affect drug choices and surgical decision-making. This may have caused considerable selection bias. Second, there may have been a risk of bias due to possible unmeasured or unknown confounding factors, including the body mass index, smoking, previous history of SSI or VTE, comorbidities, medications other than bDMARDs and degree of physical activity among RA patients. The general condition of patients and disease severity of RA were not consistent among the included studies. Third, Orthopedic surgery for RA involves a wide variety of procedures, including joint arthroplasty, fusion, artificial joints and synovectomy. Moreover, the surgical site range from upper to lower extremities. SSI and VTE are also expected to vary in susceptibility depending on the surgical site and type of surgery, and there may be a bias in lumping them all together. Fourth, the definition of SSI varies among articles, and several studies did not have a sufficient definition. It will be necessary to conduct further analyses incorporating several randomized control studies in order to obtain more reliable conclusions.

In summary, the use of bDMARDs seems to increase the rate of postoperative SSI. However, it may have different consequences for non-TNF bDMARDs. The patients treated with bDMARDs have significantly higher rate of postoperative VTE than those without bDMARD treatment. In contrast, the incidence of postoperative osseous complication could be lower in RA patients treated with bDMARDs. Based on these findings, bDMARDs have to be used with appropriate attention about postoperative SSI and VTE, and we should improve handling of bDMARDs because they might also have positive aspect in the perioperative period in patients with RA.

## Acknowledgments

We are very grateful to Dr S.K., T.I., S.K., K.M. of AKH for their kind assistance.

## Author contributions

**Conceptualization:** Takahito Suto, Koichi Okamura, Hirotaka Chikuda.

**Data curation:** Koichi Okamura, Hideo Sakane.

**Formal analysis:** Takahito Suto.

**Investigation:** Koichi Okamura, Hideo Sakane, Chisa Okura, Tetsuya Kaneko.

**Supervision:** Hirotaka Chikuda.

**Writing – original draft:** Takahito Suto.

## Supplementary Material
