# Supplementary material for: The impact of bDMARDs on postoperative complications in patients with rheumatoid arthritis: A systematic review and meta-analysis
Source: Medicine (Baltimore). 2023 Nov 24;102(47):e36132. doi: 10.1097/MD.0000000000036132 (PMC10681505; doi:10.1097/MD.0000000000036132)
Supplement: Supplementary file 1 [file medi-102-e36132-s001.docx]

**Supplementary Table 1.** Risk of bias assessment for individual studies on postoperative SSI using the ROBINS-I tool.

| **Author** | **Confounding** | **Participant selection** | **Classification of interventions** | **Deviation from intended intervention** | **Missing data** | **Measurement of outcomes** | **Selection of the**  **reported result** | **Overall** |
| --- | --- | --- | --- | --- | --- | --- | --- | --- |
| Bibbo 2004 | Serious | Moderate | Moderate | Moderate | Low | Serious | Moderate | Serious |
| Giles 2006 | Moderate | Moderate | Moderate | Moderate | Low | Moderate | Low | Moderate |
| den Broeder 2007 | Low | Low | Low | Moderate | Moderate | Low | Low | Moderate |
| Bongartz 2008 | Moderate | Low | Low | Moderate | Moderate | Low | Low | Moderate |
| Hirao 2009 | Serious | Serious | Moderate | Serious | Low | Serious | Moderate | Serious |
| Hirano 2010 | Moderate | Moderate | Moderate | Moderate | Low | Moderate | Low | Moderate |
| Kawakami 2010 | Moderate | Moderate | Moderate | Moderate | Low | Low | Low | Moderate |
| Galloway 2011 | Moderate | Moderate | Moderate | Moderate | Moderate | Low | Low | Moderate |
| Momohara 2011 | Moderate | Moderate | Low | Moderate | Moderate | Low | Low | Moderate |
| Suzuki 2011 | Serious | Serious | Serious | Serious | Serious | Serious | Moderate | Serious |
| Johnson 2013 | Serious | Serious | Moderate | Moderate | Moderate | Moderate | Serious | Serious |
| Scherrer 2013 | Serious | Serious | Moderate | Moderate | Moderate | Moderate | Moderate | Serious |
| Kubota 2014 | Serious | Serious | Moderate | Serious | Moderate | Moderate | Moderate | Serious |
| Kadota 2016 | Moderate | Moderate | Moderate | Moderate | Low | Low | Moderate | Moderate |
| Tada 2016 | Moderate | Moderate | Moderate | Moderate | Low | Moderate | Moderate | Moderate |
| Cordtz 2018 | Low | Low | Moderate | Moderate | Moderate | Moderate | Moderate | Moderate |
| Onodera 2019 | Moderate | Serious | Moderate | Moderate | Moderate | Moderate | Serious | Serious |
| Borgas 2020 | Moderate | Serious | Moderate | Moderate | Moderate | Moderate | Serious | Serious |
| Ito 2020 | Moderate | Moderate | Moderate | Moderate | Moderate | Low | Low | Moderate |
| Yamashita 2022 | Serious | Serious | Moderate | Moderate | Serious | Moderate | Serious | Serious |

ROBINS-I: Risk Of Bias In Non-randomised Studies - of Interventions; SSI: surgical site infection
